# Supplementary material for: High-intensity training enhances executive function in children in a randomized, placebo-controlled trial
Source: eLife. 2017 Aug 22;6:e25062. doi: 10.7554/eLife.25062 (PMC5566451; doi:10.7554/eLife.25062)
Supplement: Table 1—source data 1. — The plot shows the eigenvalues associated with each factor plotted against each factor, and supports the decision to retain two factors. [file elife-25062-table1-data1.docx]

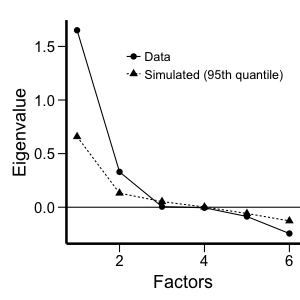


**Table 1-source data 1. Scree plot for the exploratory factor analysis on all cognitive measures.** The plot shows the eigenvalues associated with each factor plotted against each factor, and supports the decision to retain two factors.
