# Supplementary material for: High-intensity training enhances executive function in children in a randomized, placebo-controlled trial
Source: eLife. 2017 Aug 22;6:e25062. doi: 10.7554/eLife.25062 (PMC5566451; doi:10.7554/eLife.25062)
Supplement: Table 1—source data 2. — F1 (Cognitive Control) and F2 (Working Memory) refer to the factors extracted from an exploratory factor analysis on all six cognitive measures, with promax rotation (N = 287). [file elife-25062-table1-data2.docx]

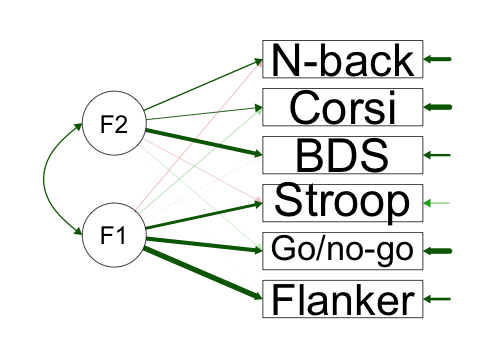


**Table 1-source data 2. Path diagram for the exploratory factor analysis on all cognitive measures.** F1 (Cognitive Control) and F2 (Working Memory) refer to the factors extracted from an exploratory factor analysis on all six cognitive measures, with promax rotation (*N* = 287).
