# Supplementary figures and images for: High-intensity training enhances executive function in children in a randomized, placebo-controlled trial
Source: eLife. 2017 Aug 22;6:e25062. doi: 10.7554/eLife.25062 (PMC5566451; doi:10.7554/eLife.25062)

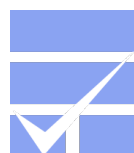

# CONSORT

TRANSPARENT REPORTING of TRIALS

## CONSORT 2010 Flow Diagram

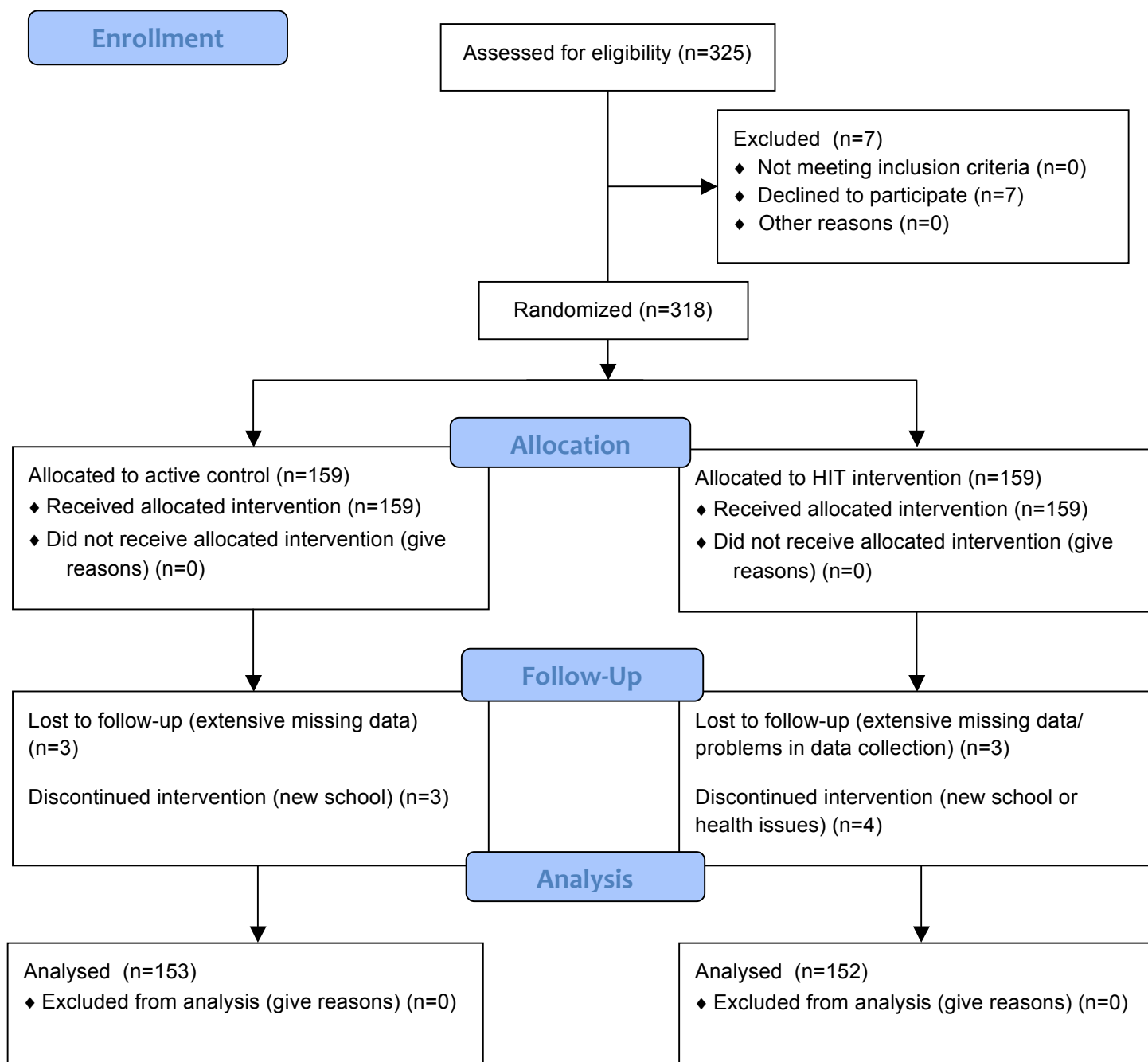

Supplement: Reporting standard 1. [file elife-25062-repstand1.pdf]
